# Accidental guidewire migration following emergency femoral central venous catheterization

**DOI:** 10.11604/pamj.2019.33.259.17043

**Published:** 2019-07-26

**Authors:** Isaac Okyere, Christiana Adu-Takyi, John Appiah Adabie, Perditer Okyere, Nana Addo Boateng

**Affiliations:** 1 Cardiovascular and Thoracic Surgery Unit, Department of Surgery, School of Medicine and Dentistry, Kwame Nkrumah University of Science and Technology/Komfo Anokye Teaching Hospital, Kumasi, Ghana; 2 Department of Child Health, Komfo Anokye Teaching Hospital, Kumasi, Ghana; 3 Renal Unit, Department of Internal Medicine, School of Medicine and Dentistry, Kwame Nkrumah University of Science and Technology and Komfo Anokye Teaching Hospital, Kumasi, Ghana; 4 Department of Anaesthesia and Intensive Care, Komfo Anokye Teaching Hospital, Kumasi, Ghana

**Keywords:** Guidewire migration, central venous catheterization, femoral vein, complication, retrieval, groin incision

## Abstract

Central venous catheter placement especially the femoral venous catheter is a common practice in critically ill patients. Awareness of potential complications of the guidewire such as guidewire migration is of utmost importance. Though potentially retrievable by a vascular surgeon or interventional radiologist if it occurs, close supervision by a senior person during passage by a junior or inexperienced person, the use of ultrasound before and after placement of catheter, and use of a checklist may help to identify and prevent its occurrence. We present a very rare complication of central venous cannulation of a guidewire migration in our institution. A 12-year-old girl presented to the Paediatric Emergency Unit (PEU) with status epilepticus and aspiration pneumonia and subsequently transferred to the Paediatric Intensive Care Unit (PICU) for ventilatory support. She had accidental guidewire migration to the left internal jugular vein following a right transfemoral central venous catheterization. She underwent successful guidewire retrieval via a right groin incision.

## Introduction

Migration of the guidewire inside the major vessel is an uncommon and serious complication of central venous cannulation with few reported cases in literature. Most cases have involved cannulation of the internal jugular or subclavian vein with migration into the right ventricle with few reports on transfemoral venous cannulation. Though potentially retrievable by a vascular surgeon or interventional radiologist if it occurs, close supervision by a senior person during passage by a junior or inexperienced person, the use of ultrasound before and after placement of catheter and use of a checklist may help to prevent its occurrence.

## Patient and observation

**Figure 1:**
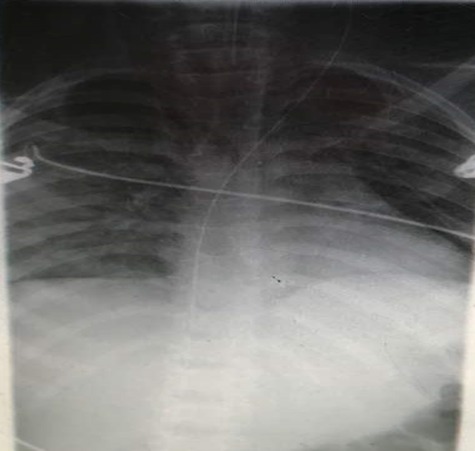
An anteroposterior chest x-ray showing the path of the guidewire through the inferior vena cava, right atrium, the superior vena cava, the innominate vein and into the left internal Jugular vein

**Figure 2:**
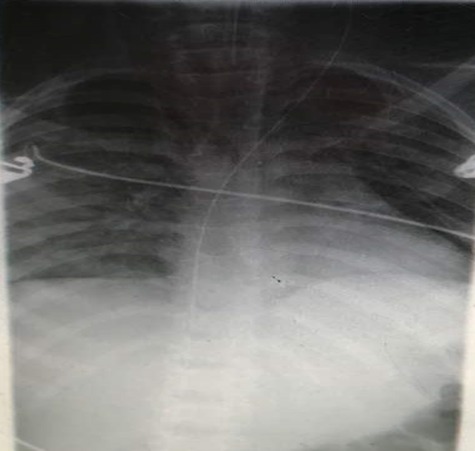
The pelvic x-ray showing the tip of the guidewire in the pelvis (right femoral vein)

Informed patient consent was obtained for this patient treatment. A 12-year old girl was referred to the paediatric intensive care unit (PICU) from the Paediatric Emergency Unit with status epilepticus and aspiration pneumonia. She had been on admission at a peripheral hospital and referred for further management on account of status epilepticus, which was refractory to most of the common antiseizure medications. She was brought to PICU for ventilatory support due to respiratory depressive effects that the additional antiseizure medications; notably high dose phenobarbitone, sodium valproate, midazolam et al. to be given were noted for. A diagnosis of Febrile Infection Related Epilepsy Syndrome (F.I.R.E.S) was entertained due to her history. She had been well prior to this and this was the first ever episode of a seizure. She had had status for over a day (more than 50 episodes) from the referral hospital and was getting worse. She had more seizures when touched and so the need to get a central venous access for adequate resuscitation and management. A resident attempted cannulation of the right femoral vein. Venous blood was aspirated and the guidewire was introduced smoothly. The cannula was removed with the guidewire in situ and the dilator was advanced to create a track. While advancing the dilator, the end of the guidewire accidently slipped from the operator's hand. On withdrawal of the dilator, the guidewire was missing. A small incision was made at the insertion site to find the end of the guidewire in the subcutaneous tissue, but this was unsuccessful. A mobile chest X-ray revealed the guidewire has traversed the inferior vena cava, the right atrium, the superior vena cava, the left innominate vein and lodged into the left internal jugular vein as shown in Figure 1. The venous access was done by a second year resident supervised by an experienced emergency physician. The cardiothoracic surgeon was consulted. Patient was evaluated and prepared for exploration and retrieval via a groin incision as the tip of the guidewire was found below the inferior ramus of the right pelvic bone from the pelvic x-ray shown in Figure 2.

**Figure 3:**
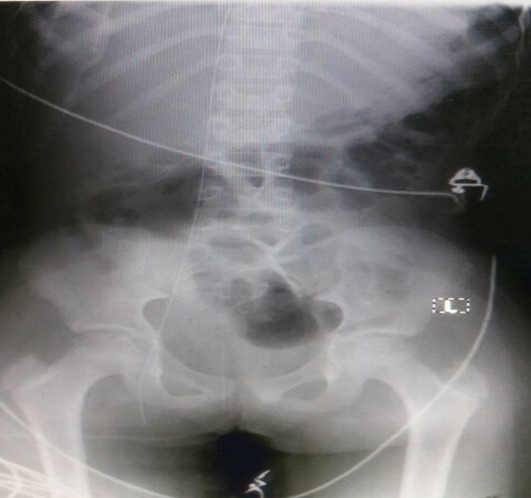
Showing the right femoral vein with the guidewire

**Operation:** under general anaesthesia and supine position, a right longitudinal incision was made over the right femoral vein. The right femoral vein was dissected free and isolated on a sling. A longitudinal venotomy: incision on the vein was made and the guidewire which was visible was carefully removed with an artery forceps as shown in Figure 3. The venotomy was repaired primarily with a 6/0 prolene suture and the wound closed up in layers. She recovered uneventfully from surgery.

**Postoperative course:** she however developed uncontrollable sepsis 3 weeks from the aspiration pneumonia, further complicated by uncontrolled seizures from the suspected F.I.R.E.S and died after 7 weeks on admission.

## Discussion

Central venous placement is a common technique in emergency and critical care for many reasons such as fluid administration, blood sampling, Central Venous Pressure (CVP) monitoring, and the administration of vesicant medications having pH greater than 9.0 or less than 5.0, osmolality greater than 500 mOsm, parenteral nutrition with dextrose concentration greater than 10%, intravenous inotropes, and for long-term intravenous therapy over weeks, months or years. Although it is not a very difficult procedure, severe complications sometimes occur. Reported complication rate of central venous cannulation ranges from 0.3-12% according to Mansfield *et al.* [1] and they often depend on the experience level of the physician [2]. Potential complications include failure to locate or cannulate the vein, puncture of the subclavian artery, common carotid artery, and femoral artery depending on the site of venous cannulation, pneumothorax, mediastinal hematoma, hemothorax, and injury to adjacent nerves. Seldinger originally described his technique using a guidewire in 1953 [3], and complications were soon to follow.

**The Seldinger method:** involves gaining access to the central vein via an introducer needle through which a matching guidewire is threaded to maintain venous access after needle withdrawal. The catheter is advanced into position over the intravascular guidewire, which is subsequently removed from the catheter. Loss or migration of the guidewire is a serious and potentially life-threatening complication with reports of fatalities in up to 20% of cases when the complete wire is lost [4]. The central venous options for central line cannulation include the subclavian vein, the internal jugular vein and the femoral vein, the basilic and the cephalic veins. The femoral vein is the most common route for insertion of central lines in emergency, as it has one of the lowest rates of complications. There is no risk of pneumothorax and in cases with coagulopathie; it is relatively easy to apply compression when bleeding ensues. But without ul-trasound, some complications can be observed, like failure, arterial puncture and haematoma formation which then increases the risk of infection and thrombosis. The complications associated with the guidewire, like failure to pass, loss in the vessel, kinking, knotting and breakage were rare with this route with few reported cases of guidewire migration or loss following cannulation [5,6]. Cheddie *et al.* in 2013 [7], reviewed their two cases of guidewire migration where they used interventional percutaneous technique to retrieve them.

They therefore proposed that open technique of lost guidewire retrieval by a vascular surgeon should only be employed when the interventional percutaneous method had failed. In addition, in the case of failed guidewire retrieval by any method, the retained guidewire in-situ should be managed by prolonged anticoagulation and prophylactic antibiotics, however they failed to clearly define the duration of this therapy [7]. Huang *et al.* also described similar method of guidewire retrieval by interventional technique by an interventional cardiologist [5]. However similar to our case, Braham *et al.* described an open surgery technique to remove lost guidewire in the right femoral vein which was done by a vascular surgeon [6]. Similar approach via a groin incision to retrieve the guidewire was used in these two cases, since in both cases the proximal tip of the guidewire was still in the femoral vein. Omar *et al.* also described how a lost guidewire they had passed for a patient using the internal jugular was removed by the vascular surgical team using open surgical technique [8]. Therefore though, the management of guidewire embolism/loss or migration is poorly defined in the literature, Cheddie *et al.* [7] recommend that in the case of a superficial guidewire embolus in which the guidewire can be palpated in the vessel (internal jugular vein/femoral vein), a venous cutdown (open approach or technique),can be performed to extract the guidewire, however if facilities are available then interventional radiologic techniques such as the use of Dormia basket, gooseneck snare, endovascular forceps can be employed to percutaneously extract the guidewire [9]. If the guidewire has embolised systemically, a chest x-ray must be performed to determine the position of the guidewire [7]. The complications of leaving guidewire in-situ are thrombosis, infections, post-phlebitic syndrome, pulmonary embolism, and arrhythmias, cardiac and vascular damage [10]. However, this complication may remain unnoticed for a significant period of time [2]. As far as we know this is the first reported case in Ghana and in Africa.

## Conclusion

Central venous catheter placement especially femoral central venous catheterization is a common practice in critically ill patients. Awareness of the rare complication of guidewire migration or loss is of utmost importance. Migrated guidewire can be retrieved or removed by either a vascular surgeon or an interventional radiologist. However close supervision by a senior person during central venous catheterization by a junior or inexperienced person, the use of ultrasound before and after placement of the catheter, and use of a checklist may help to identify and prevent its occurrence.

## Competing interests

The authors declare no competing interests.
